# Decomposition and Mineralization of Dimethyl Phthalate in an Aqueous Solution by Wet Oxidation

**DOI:** 10.1155/2015/164594

**Published:** 2015-07-05

**Authors:** Dar-Ren Ji, Chia-Chi Chang, Shih-Yun Chen, Chun-Yu Chiu, Jyi-Yeong Tseng, Ching-Yuan Chang, Chiung-Fen Chang, Sheng-Wei Chiang, Zang-Sie Hung, Je-Lueng Shie, Yi-Hung Chen, Min-Hao Yuan

**Affiliations:** ^1^Graduate Institute of Environmental Engineering, National Taiwan University, Taipei 106, Taiwan; ^2^Department of Cosmetic Science and Application, Lan Yang Institute of Technology, Yilan 261, Taiwan; ^3^Department of Chemical Engineering, National Taiwan University, Taipei 106, Taiwan; ^4^Department of Environmental Science and Engineering, Tunghai University, Taichung 407, Taiwan; ^5^Chemical Engineering Division, Institute of Nuclear Energy Research, Atomic Energy Council, Lungtan, Taoyuan 325, Taiwan; ^6^Department of Environmental Engineering, National Ilan University, Yilan 260, Taiwan; ^7^Department of Chemical Engineering and Biotechnology, National Taipei University of Technology, Taipei 106, Taiwan

## Abstract

Dimethyl phthalate (DMP) was treated via wet oxygen oxidation process (WOP). The decomposition efficiency *η*_DMP_ of DMP and mineralization efficiency *η*_TOC_ of total organic carbons were measured to evaluate the effects of operation parameters on the performance of WOP. The results revealed that reaction temperature *T* is the most affecting factor, with a higher *T* offering higher *η*_DMP_ and *η*_TOC_ as expected. The *η*_DMP_ increases as rotating speed increases from 300 to 500 rpm with stirring enhancement of gas liquid mass transfer. However, it exhibits reduction effect at 700 rpm due to purging of dissolved oxygen by overstirring. Regarding the effects of pressure *P*_*T*_, a higher *P*_*T*_ provides more oxygen for the forward reaction with DMP, while overhigh *P*_*T*_ increases the absorption of gaseous products such as CO_2_ and decomposes short-chain hydrocarbon fragments back into the solution thus hindering the forward reaction. For the tested *P*_*T*_ of 2.41 to 3.45 MPa, the results indicated that 2.41 MPa is appropriate. A longer reaction time of course gives better performance. At 500 rpm, 483 K, 2.41 MPa, and 180 min, the *η*_DMP_ and *η*_TOC_ are 93 and 36%, respectively.

## 1. Introduction

Phthalic acid esters (PAEs) including dimethyl phthalate (DMP) are major plasticizer to improve the mechanical properties of polymers. These polymers in turn were used for making tableware such as forks, spoons, dishes, cups, and lunchboxes. In fact, the PAEs are added via noncovalent bonding with the polymers. It means PAEs are easily released to the hot soup, heated food, and oily contents from the tableware and are orally ingested daily [1, 2].

PAEs are endocrine disrupter substances (EDSs) too. Their derivatives exhibit the similar structure with endocrine of human and other animals, thus inducing the possibility of cancer of human and the sex development of male. The worst influence of EDSs to the ecosystem would be extinction for endanger species [3, 4].

Although PAEs can be effectively removed from the aqueous phase by adsorption [5] which also has been applied to treat other EDSs [6, 7], it needs the regeneration of exhausted adsorbent and the post treatment of concentrated regeneration solution. Activated sludge based biological sewage treatment system needs 20 d to reach 71% mineralization efficiency and is not beneficial to deal with toxic DMP. It was biodegraded to monomethyl phthalate (MMP) and phthalic acid (PA) after treatment of 2.5 d [8–10]. Some advanced solutions were proposed such as photolysis [11–13], photocatalysis [11–15], electrochemical [16, 17], and oxidant-added oxidation [11, 14, 18, 19] methods. Most of these treatments need postbiological process to further mineralize the decomposed compounds. The unconsumed oxidant residue needs to be neutralized for matching the effluent standard [14, 15]. Processes of wet air oxidation (WAP) and wet oxygen oxidation (WOP) with (CWAP and CWOP) and without catalysts have been successfully employed for oxidation treatments [20–30]. For example, in a study on the treatment of high-strength industrial wastewater, Lin and Ho [27] reported that the chemical oxygen demand (COD) removal efficiencies (*η*_COD_) via WAP, WOP, and CWAP with CuSO_4_ catalyst were 65, 73, and 75%, respectively, at 473 K, 3 MPa, 300 rpm, 1 L/min gas flow rate, and 2 h. The application of WAP and WOP has the advantages avoiding the posttreatment of unwanted residual oxidant species and no need for the recovery and regeneration of catalyst, compared with oxidant-added oxidation and catalytic oxidation, respectively. The abundant dissolved oxygen left can improve the performance of regular biological sewage system if needed [18]. Moreover, WOP gives *η*_COD_ only slightly less than catalytic oxidation while higher than WAP. This study thus employed WOP to treat the DMP-containing aqueous solution.

## 2. Experimental Materials and Methods

### 2.1. Materials

DMP with purity of 99.5% was supplied by Hayashi Pure Chemical Industries Ltd. (Osaka, Japan). The mobile phase of high performance liquid chromatography (HPLC) was composed as acetonitrile (CH_3_CN) : DI water = 1 : 1, where acetonitrile of 100% purity was from J. T. Baker, Phillipsburg, NJ. The solvent for apparatus cleaning is acetone (C_3_H_4_O) with purity of 99.5% by Mallinckrodt Chemicals, St. Louis, MD. The reagents for measurement of total organic carbon (TOC) were (1) carrier gas: 99.99% N_2_ from San Fu Chemical Co. Ltd., Taipei, Taiwan; (2) oxidant: sodium peroxydisulfate, Na_2_S_2_O_8_ (99% purity), from Nacalai Tesque, Kyoto, Japan; (3) standard solution: anhydrous potassium biphthalate, KHP, C_8_H_5_KO_4_ (99.0% purity), from Riedel-de Haën, Seelze, Germany. The reaction gas O_2_ (99.99% purity) and air (O_2_ : N_2_ = 20 : 80, 99.99% purity) were purchased from San Fu Chemical Co. Ltd., Taipei, Taiwan.

### 2.2. Methods

**Figure 1:**
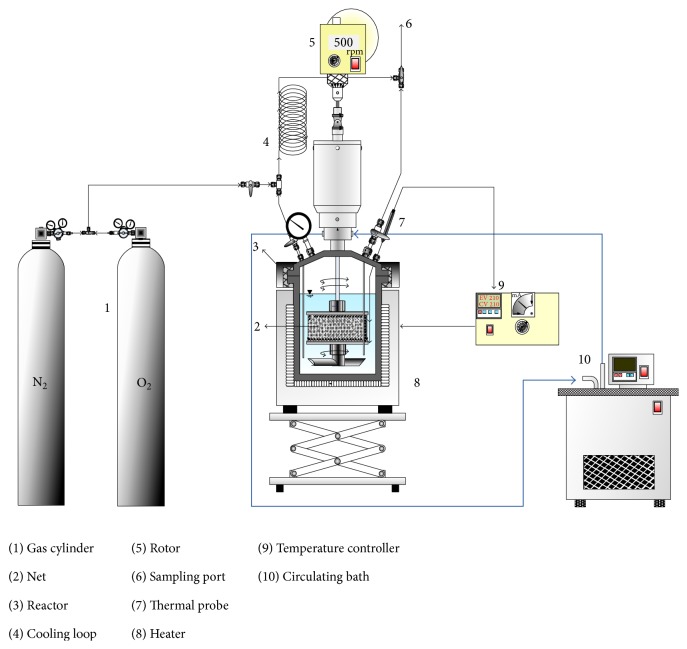
Schematic diagram of wet oxygen oxidation system.

The pressurized autoclave reaction system is shown in Figure 1. A 1 L bench top reactor is used. It is made of stainless steel 316 and equipped with a stirring rotor (DC-2RT44, Hsing-Tai Machinery Ind. Co., Taipei, Taiwan), pressure display module, and K-type thermal couple. The temperature of heater (Model-TC-10A, Macro Fortunate, Taipei, Taiwan) is controlled with temperature controller (Model-BMW-500, Newlab Instrument Co., Taipei, Taiwan). Mass flow controller of Model 5850E manufactured by Brooks (Hatfield, PA) is employed to control the gas flow rate. The bearing is cooled by cooling water from circulating bath (Model-B403, Firstek Scientific, Taipei, Taiwan). The upper cap of vessel has six holes with five for two sampling valves, thermal couple, pressure gauge, and release valve, while one for spare port. The experiments were batch type with volume of liquid of DMP solution (*V*_*L*_) of 400 mL. The sampling valves are connected to cooling coil. The pressured vapor was captured to the coil and then cooled while keeping the pressure of the reactor. After 5 mL liquor was sampled, the noncollected cooled liquid was conducted back to the reactor.

The initial concentration (*C*_0_) of DMP solution was 100 mg/L. The concentrations of DMP of samples (*C*) were analyzed by high performance liquid chromatography (HPLC, Viscotek Model 500, Houston, TX), while those of total organic carbon (TOC) were analyzed by TOC analyzer (Model 1010, O.I. Analytical, NY). The column of HPLC is 516C-18 of 25 cm  ×  4.6 mm with ID 5 *μ*m (Supelco Inc., Bellefonte, PA). The TOC analyzer uses nondispersive infrared (NDIR) detector, with carrier gas of N_2_, oxidative agent of 10% sodium peroxydisulfate solution, and TOC standard solution of anhydrous potassium biphthalate. The precision of experimental data was indicated in figures by error bar with standard deviation (*σ*_*n*−1_) above and below the average value.

The batch WOP process was performed in two stages. The first is heating stage. The DMP-containing solution, which was prebubbled by N_2_ to purge out the residual oxygen, was filled into the autoclave reactor and then heated from room temperature 283 K to the set reaction temperature (*T*) without any oxidant. The tested temperatures were 463, 473, and 483 K. The initial time (*t*) was noted as 0_*i*_, while the final time of the first stage as 0_*f*_. In the second stage, the working gas O_2_ was introduced into the reactor at *t* = 0_*f*_ to the desired operation pressure (*P*_*T*_) to continue the oxygen oxidation reaction.

The major operation parameters of batch WOP were examined including (1) the stirring speed (Nr), (2) reaction temperature *T*, and (3) operation pressure *P*_*T*_. The initial pH value (pH_0_) was not adjusted while reflected by the *C*_0_. Values of parameters are listed in Table 1 referring to those of others [27, 29]. For example, Lin and Ho [27] performed the experiments with Nr = 100–400 rpm, *P*_*T*_ = 2.5–5.0 MPa, and *T* = 423–513 K. They reported that (1) 300 rpm and 3 MPa were appropriate and (2) *T* was the most important operation variable with marginal enhancing effect for *T* above 498 K. The present study extended Nr to 500–700 rpm, while it employed *P*_*T*_ and *T* in the proper ranges of those of Lin and Ho [27].

## 3. Results and Discussion

### 3.1. Effects of Rotation Speeds Nr

**Figure 2:**
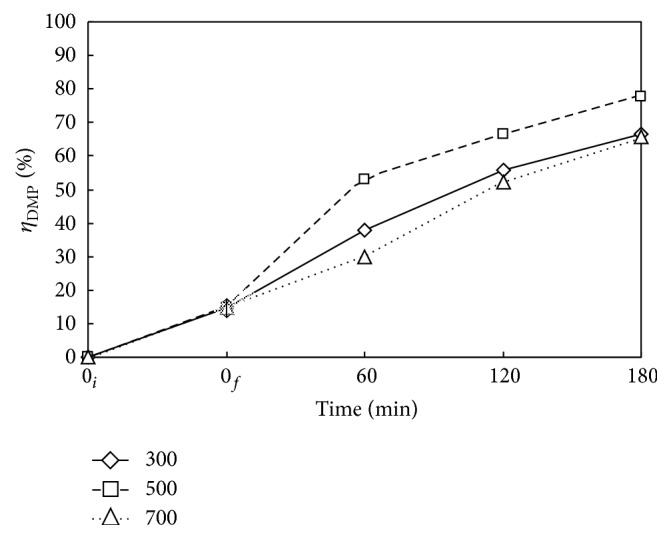
Time variation of decomposition efficiency of DMP (*η*_DMP_) via WOP at various rotating speeds Nr. ⋄, □, and △: Nr = 300, 500, and 700 rpm. *C*_0_ = 100 mg L^−1^, *V*_*L*_ = 400 mL, *T* = 473 K, and *P*_*T*_ = 2.41 MPa. Working gas after time = 0_*f*_ is O_2_. ↕: Mean and Standard deviation (SD, *n* − 1 method) at *t* = 0_*f*_: 14.8 ± 2.8.

Figure 2 illustrate the variation of decomposition efficiency of DMP (*η*_DMP_) with reaction time *t* at various rotation speeds (Nr = 300, 500, and 700 rpm). Other conditions are reaction temperature *T* = 473 K and operation pressure *P*_*T*_ = 2.41 MPa. As expected, more DMP is decomposed with longer *t* giving higher *η*_DMP_. The *η*_DMP_ is 66, 78, and 66% at *t* = 180 min for Nr = 300, 500, and 700 rpm, respectively. In general, a good gas liquid mixing assists the reaction. Thus, an increase of Nr from 300 to 500 rpm increases the gas liquid mass transfer and offers a higher *η*_DMP_. However, the dissolved oxygen needed for reaction may be tripped or purged out from liquid to gas as further increasing the Nr, say to 700 rpm, reducing the *η*_DMP_. The Nr of 500 rpm leads to better increasing trend of *η*_DMP_.

It is noted that although the effects of Nr of low rpm, say below 300 rpm, on the system performance were not investigated in this study, its qualitative effects may be realized referring to the work of Lin and Ho [27] dealing with the treatment of high-strength industrial wastewater. They examined the effects of Nr from 100 to 400 rpm on the chemical oxygen demand removal efficiencies *η*_COD_, indicating apparently significant effect as Nr below 300 rpm. An Nr of 300 rpm was thus adopted for their further experiments. This thus justified the adoption of 500 rpm for the followed experiments of the present study, assuring the good mixing.

**Figure 3:**
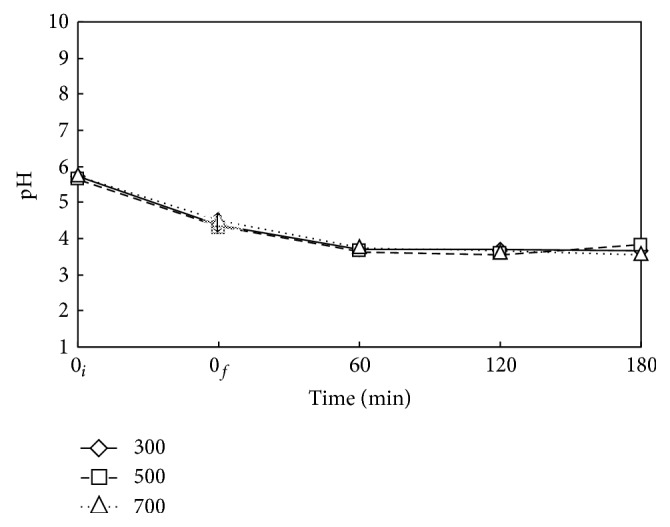
Time variation of pH value for the decomposition of DMP via WOP at various Nr. ⋄, □, and △: Nr = 300, 500, and 700 rpm. *C*_0_ = 100 mg L^−1^, *V*_*L*_ = 400 mL, *T* = 473 K, and *P*_*T*_ = 2.41 MPa. Working gas after time = 0_*f*_ is O_2_. ↕: Mean and Standard deviation (SD, *n* − 1 method) at *t* = 0_*f*_: 4.4 ± 0.1.

The effect of reaction time on the pH value of DMP-containing solution during WOP at different Nr is depicted in Figure 3. The decrease of pH value as oxidation decomposition takes place indicates the formation of acidic products. Although the decompositions are significant from 60 to 180 min as shown in Figure 2, the pH value stays nearly the same at about 4 after 60 min. This might be due to the cause that some intermediate acidic products from the decomposition of DMP are further broken down to small acidic fragments of low solubility being released to gas phase, leaving the pH value of liquid essentially not altered for *t* longer than 60 min. The negligible effect of Nr on pH value as Nr is sufficiently high as 300 rpm or higher might be attributed to the balance of enhancement of gas liquid mass transfer and the purge of small acidic fragments by rotation stirring.

### 3.2. Effects of Reaction Temperature *T*

**Figure 4:**
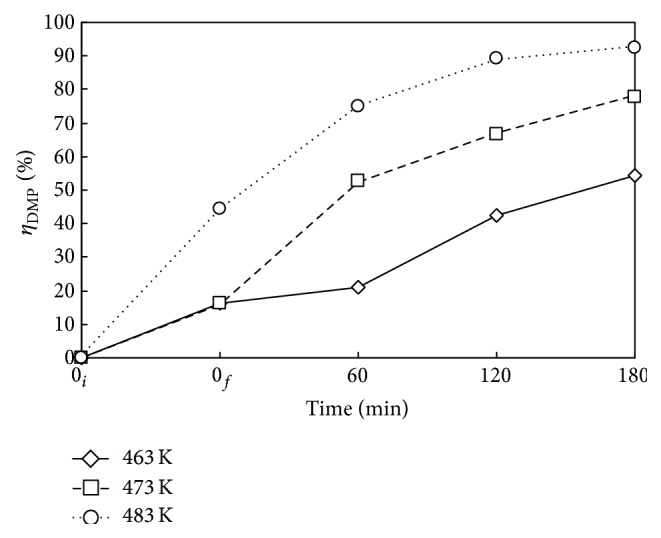
Time variation of *η*_DMP_ via WOP at various temperatures *T*. ⋄, □, and ○: *T* = 463, 473, and 483 K. *C*_0_ = 100 mg L^−1^, *V*_*L*_ = 400 mL, *P*_*T*_ = 2.41 MPa, and Nr = 500 rpm. Working gas after time = 0_*f*_ is O_2_.

**Figure 5:**
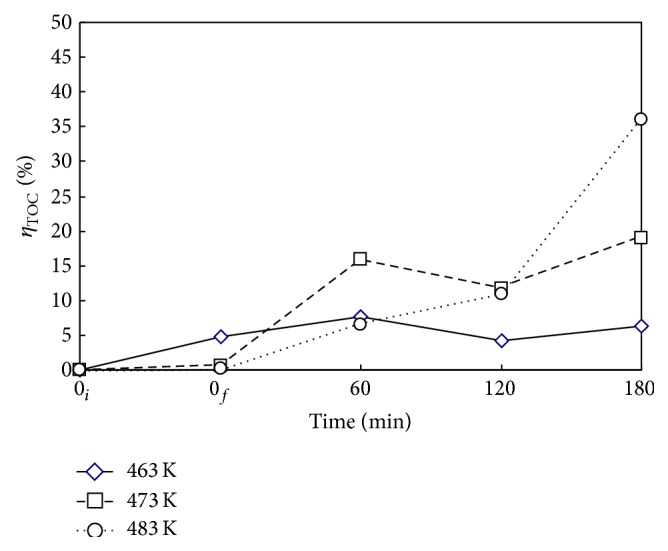
Time variation of mineralization efficiency of DMP (*η*_TOC_) via WOP at various *T*. ⋄, □, and ○: *T* = 463, 473, and 483 K. *C*_0_ = 100 mg L^−1^, *V*_*L*_ = 400 mL, *P*_*T*_ = 2.41 MPa, and Nr = 500 rpm. Working gas after time = 0_*f*_ is O_2_.

Figures 4 and 5 show the time variations of *η*_DMP_ and *η*_TOC_ at reaction temperatures *T* of 463, 473, and 483 K for the case with Nr = 500 rpm and *P*_*T*_ = 2.41 MPa. In the heating period from 0_*i*_ to 0_*f*_ without oxidant, DMP underwent mainly the hydrothermal decomposition accompanied with slight mineralization. The *η*_DMP_ is 17% for 463 and 473 K while 45% for 483 K at the end of heating period with no oxygen. The decomposition of DMP is very vigorous at high temperature. But the *η*_TOC_ is lower than 10% for all three temperatures because of the oxidant lack. With the presence of oxygen, the *η*_DMP_ was greatly enhanced while *η*_TOC_ moderately improved. The results indicated the low reactivity of acidic product fragments with oxygen. As expected, both *η*_DMP_ and *η*_TOC_ increased as reaction time and temperature increased. At *T* = 483 K and *t* = 180 min, the *η*_DMP_ and *η*_TOC_ were 93 and 36%, respectively.

**Figure 6:**
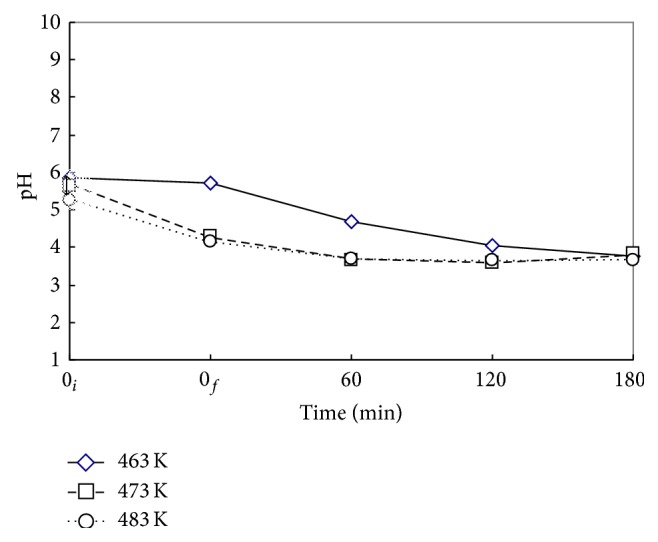
Time variation of pH value for the decomposition of DMP via WOP at various *T*. ⋄, □, and ○: *T* = 463, 473, and 483 K. *C*_0_ = 100 mg L^−1^, *V*_*L*_ = 400 mL, *P*_*T*_ = 2.41 MPa, and Nr = 500 rpm. Working gas after time = 0_*f*_ is O_2_. ↕: Mean and Standard deviation (SD, *n* − 1 method) at *t* = 0_*i*_: 5.6 ± 0.3.

Figure 6 demonstrates the variation of pH value with time at various temperatures. As in Figure 3, the pH value decreased with time, while it levels off at a longer time depending on the temperature, for example, at 60 min for higher temperatures of 473 and 483 K while at 120 min for lower temperature of 464 K. Thus, a higher temperature case promotes the decomposition reaction, generally lowering and leveling the pH value faster than the lower temperature case. For 483 K, the pH value decreases to a leveling value of around 4 after 60 min.

### 3.3. Effects of Operation Pressure *P*_*T*_

**Figure 7:**
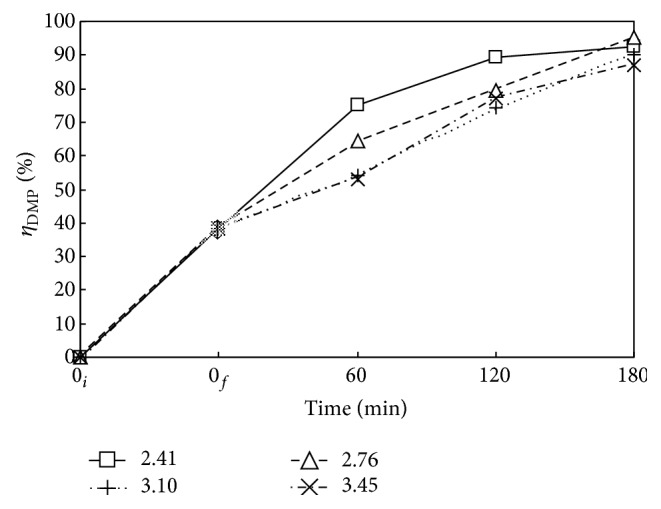
Time variation of *η*_DMP_ via WOP at various pressures. □, △, +, and ×: *P*_*T*_ = 2.41, 2.76, 3.10, and 3.45 MPa. *C*_0_ = 100 mg L^−1^, *V*_*L*_ = 400 mL, *T* = 483 K, and Nr = 500 rpm. Working gas after time = 0_*f*_ is O_2_. ↕: Mean and Standard deviation (SD, *n* − 1 method) at *t* = 0_*f*_: 38.2 ± 5.3.

**Figure 8:**
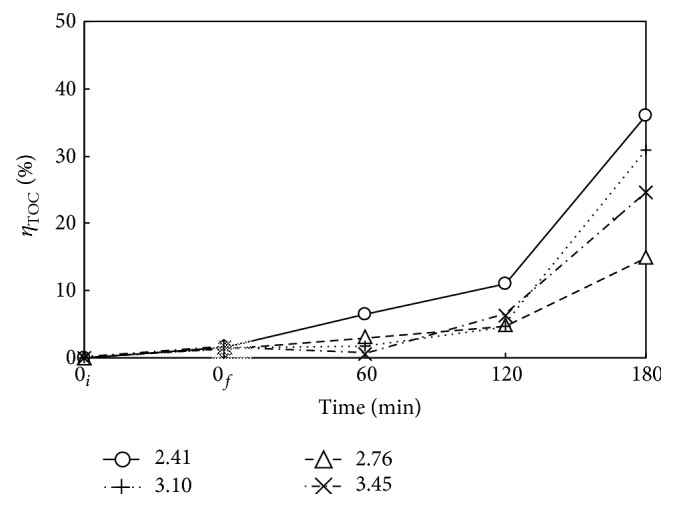
Time variation of *η*_TOC_ via WOP at various pressures. ○, △, +, and ×: *P*_*T*_ = 2.41, 2.76, 3.10, and 3.45 MPa. *C*_0_ = 100 mg L^−1^, *V*_*L*_ = 400 mL, *T* = 483 K, and Nr = 500 rpm. Working gas after time = 0_*f*_ is O_2_. ↕: Mean and Standard deviation (SD, *n* − 1 method) at *t* = 0_*f*_: 1.5 ± 1.3.

Figures 7 and 8 present the *η*_DMP_ and *η*_TOC_ versus time at *P*_*T*_ of 2.41, 2.76, 3.10, and 3.45 MPa with Nr = 500 rpm and *T* = 483 K. Both *η*_DMP_ and *η*_TOC_ increase with time as expected. The oxygen was filled to reach the desired pressure right after heating period, that is, at *t* = 0_*f*_. There is no oxidant in the time period from 0_*i*_ to 0_*f*_. The DMP is hydrothermally decomposed in heating period, giving *η*_DMP_ of around 33 to 45%. The DMP is only slightly mineralized with low *η*_TOC_ of about 0.3 to 3.1%. In the presence of oxygen, both *η*_DMP_ and *η*_TOC_ are enhanced as decomposition and mineralization proceed. The oxidative decomposition of DMP essentially consists of two-stage reversible reactions as illustrated in Figure 10, which is discussed in the next section. The decomposition of DMP and intermediates to short-chain aliphatic acid and then CO_2_ are proposed by referring to the mechanism for the ozonation of DMP with UV and catalyst presented by Chang et al. [11]. An increase of oxygen as well as temperature enhances the forward reactions toward mineralization way, while the accumulation of CO_2_ reversely inhibits the mineralization according to Le Chatelier's principle [31]. Thus, sufficient oxygen with satisfactorily high *P*_*T*_ is needed to ensure the forward oxidative decomposition reaction of DMP. For example, *P*_*T*_ at 2.41 MPa yields *η*_DMP_ and *η*_TOC_ of 93 and 36% at 180 min, respectively. Although higher *P*_*T*_ with more oxygen favors the forward decomposition reaction of DMP by oxygen, the absorption of accumulated gaseous products such as CO_2_ and decomposed short-chain hydrocarbon fragments in the closed reaction system increases as *P*_*T*_ increases. The reabsorption of gaseous products back into the solution thus inhibits the forward reaction. Hence, as indicated in Figures 7 and 8, *P*_*T*_ of 2.41 MPa is more appropriate than those of 2.76 to 3.45 MPa.

**Figure 9:**
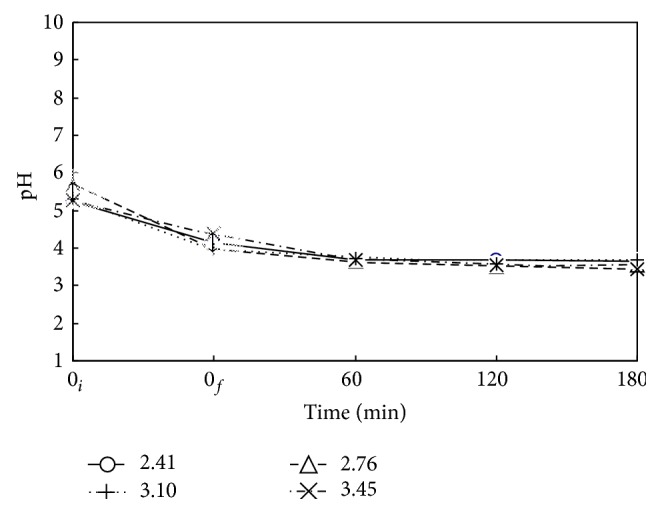
Time variation of pH value for decomposition of DMP via WOP at various pressures. ○, △, +, and ×: *P*_*T*_ = 2.41, 2.76, 3.10, and 3.45 MPa. *C*_0_ = 100 mg L^−1^, *V*_*L*_ = 400 mL, *T* = 483 K, and Nr = 500 rpm. Working gas after time = 0_*f*_ is O_2_. ↕: Mean and Standard deviation (SD, *n* − 1 method) at *t* = 0_*i*_: 5.2 ± 0.2 and at *t* = 0_*f*_: 4.1 ± 0.2.

Figure 9 plots pH value versus time at various *P*_*T*_. The reduction of pH value in hydrothermal decomposition period is more vigorous than that in the oxidative decomposition period. The trend is similar to that of Figure 3 previously discussed. The increase of *P*_*T*_ higher than 2.41 MPa exhibits negligible effect on the pH value. The pH value levels off, indicating the limited oxidative mineralization to CO_2_ and the gas liquid absorption balance of acidic compounds of CO_2_ and decomposed short-chain hydrocarbon fragments.

It is noted that the *P*_*T*_ was the sum of partial pressures of oxygen (*P*_O_2__) and water vapor (*P*_WV_). The saturation *P*_WV_ varies with temperature and is about 2.3 MPa at 483 K [27]. Setting *P*_*T*_ at 2.41 and 3.45 MPa gave *P*_O_2__ of 0.11 and 1.15 MPa, respectively, for supplying the oxygen for mineralization reaction. Referring to the study of Lin and Ho [27] using 2.5 MPa as the lowest setting at 473 K, this analysis thus did not employ *P*_*T*_ lower than 2.41 MPa at 483 K.

### 3.4. Mechanism of Two-Stage Decomposition of DMP via WOP

**Figure 10:**
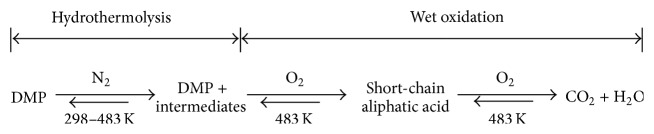
Two stages for the decomposition of DMP via WOP.

In this test, the reactions are involved in components of DMP, oxygen, intermediate products, and ultimate end products of CO_2_ and H_2_O. The intermediates are the decomposed short-chain hydrocarbon fragments which are acidic as reflected by the low pH value. Accordingly, the mechanism of two-stage decomposition of DMP via WOP may be depicted in Figure 10. In the heating stage without oxygen, DMP is essentially hydrothermally decomposed to acidic fragments lowering the pH value with significant *η*_DMP_, while forming little CO_2_ with low *η*_TOC_. With the introduction of oxygen in the second stage, oxidation of DMP and its decomposed fragments takes place, destructing them into short-chain acids such as aliphatic acids or more completely to CO_2_ and H_2_O. The produced CO_2_, however, was kept within the closed-batch reaction system in this study.

The stoichiometry equation for the forward oxidation reaction of DMP can expressed as follows:

(1) $$\begin{array}{c} C_{10}H_{10}O_{4}+10.5O_{2}⟶10CO_{2}+5H_{2}O \end{array}$$

For complete mineralization of DMP, each mole DMP consumes 10.5 moles of O_2_ while producing 10 moles of CO_2_. The CO_2_ partial pressure contributed from the complete mineralization of DMP is about 0.045 MPa by consuming 0.047 MPa O_2_. This reaction reduces the total pressure slightly. In fact, the oxygen is not a limited factor because the minimum pressure applied is 2.41 MPa exceeding the need. However, the mineralization of reaction (1) is hindered by the accumulation of product CO_2_ in the closed-batch reaction system. It forces the backward reaction of reaction (1) according to Le Chatelier's principle [31]. The equilibrium balance of the forward and backward reaction thus limits the complete mineralization of DMP. A release of CO_2_ gas out from the reaction system would certainly assist approaching the complete mineralization of DMP.

### 3.5. Comparison with Results of Others

Comparison of the results of this study with others is illustrated in Table 2. The present WOP can reach *η*_DMP_ of 93% as high as the advanced methods (AMs) of electrochemical oxidation, photocatalytic degradation, and photocatalytic ozonation. The *η*_TOC_ of WOP of 36% is lower than those of the aforementioned AMs at some conditions, however, comparable at other conditions. It is noted that the WOP simply uses oxygen with demand of the thermal energy, while other AMs need to employ chemical agents, catalysts, and ozone along with electric or UV energies. Thus, the WOP is comparatively simple to apply. The discrepancy of incomplete mineralization of WOP may be consummated with the postbiological treatment if necessary [20]. The predecomposition of DMP by WOP certainly greatly enhances the followed biological processing.

## 4. Conclusions

This study treated the toxic endocrine disrupter substance (EDC) of DMP via wet oxidation using oxygen (WOP) without other oxidant additives, being beneficial to the subsequent biological process if necessary, while avoiding the treatment of unwanted oxidant residues. The WOP effectively decomposed the DMP, indicating its feasible application for the treatment of other EDCs.

Among the three factors investigated, namely, rotation speed Nr, reaction temperature *T*, and operation pressure *P*_*T*_, the effects of *T* are most significant. The proper conditions found are at 483 K, 2.41 MPa, and 500 rpm. The *η*_DMP_ and *η*_TOC_ of 93% and 36%, respectively, can be achieved at 180 min. The produced CO_2_ kept in the closed-batch reaction system seems to resist the further mineralization reaction from intermediates. The application of sequential release of CO_2_ while addition of O_2_ to improve the *η*_TOC_ is thus suggested.

## Figures and Tables

**Table 1:** Operation parameters and range of WOP.

Parameter of operation	Operation range
Rotation speed Nr, rpm	300, 500, 700
Temperature *T*, K	463, 473, 483
Pressure *P*_*T*_, MPa	2.41, 2.67, 3.10, 3.45
Working gas of O_2_	Pure O_2_

**Table 2:** Comparison with some results of others for the decomposition of DMP via various methods.

Study	Method	Result
Bauer et al. [1]	Anaerobic process in field municipal landfill leachates	DMP was completely hydrolysis to phthalic acid but no cleavage for aromatic ring at different pH values

Wang et al. [16]	Electro-Fenton methods by electrodes: traditional graphite cathode (G), carbon nanotube sponge (CNTS), and graphite gas diffusion electrode (GDE)	*η*_TOC_: G, 15%; GDE, 35%; CNTS, 75%

Souza et al. [17]	Electrochemical oxidation on F-doped Ti/*β*-PbO_2_ anode in filter press reactor	DMP was completely decomposed under electrolyte Na_2_SO_4_ and low current densities (10 mA), *η*_TOC_ = 25%

Chang et al. [11]	Catalytic ozonation (OZ) in high-gravity rotating packed bed (HG) with catalyst (Pt/-Al_2_O_3_) and ultraviolet (UV) (mix of UV-C, UV-B, and UV-A with 200–280, 280–315, and 315–400 nm and with intensities of 3.73, 1.59, and 3.99 W m^−2^)	*η*_DMP_ at 50 min: near 100% for Pt-OZ and UV-Pt-OZ *η*_TOC_ at 1 h: 45% (OZ); 56% (UV-OZ); 57% (Pt-OZ); 68% (UV-Pt-OZ)

Chen et al. [13]	Photocatalytic degradation using magnetic poly(methyl methacrylate) (mPMMA) and UV 254 nm	*η*_DMP_ at 4 h: 55–100% via TiO_2_/mPMMA (C1); 68–100% via Pt-TiO_2_/mPMMA (C2) *η*_TOC_ at 4 h: 7.5–37.5 % via C1; 11–64% via C2

Chen et al. [19]	Photocatalytic ozonation using TiO_2_, Al_2_O_3_, and TiO_2_/Al_2_O_3_ catalysts	*η*_DMP_ at 30 min: 2–22% without O_3_, 90–100% with O_3_. *η*_TOC_: 16–93%, 32–97% at 1, 4 h

Chen et al. [12]	Photocatalysis using magnetic Pt-TiO_2_/mPMMA	UV 185 nm contributes better removal efficiency than UV 254 nm

This study	Wet oxygen oxidation	*η*_DMP_ and *η*_TOC_ are 93 and 36% at Nr = 500 rpm, *T* = 483 K, *P*_*T*_ = 2.41 MPa, and *t* = 180 min
